# Outcomes of keraring implantation for high regular astigmatism in non-ectatic corneas: A prospective pilot case series

**DOI:** 10.1038/s41598-026-57128-7

**Published:** 2026-06-17

**Authors:** Amr Mounir, Elshimaa A Mateen Mossa, Alaa Mahmoud

**Affiliations:** Ophthalmology Department, Faculty of Medicine, Sohag University, Sohag, Egypt

**Keywords:** Diseases, Health care, Medical research

## Abstract

This study aimed to evaluate the effectiveness of intracorneal ring segments (Keraring) implantation for the management of high regular astigmatism (> 3.00 D) in eyes with non-ectatic corneas. Twenty eyes of twenty patients with high regular astigmatism and topographically normal corneas. All eyes underwent femtosecond laser–assisted implantation of SI-5 Keraring segments. Comprehensive preoperative and postoperative assessments included uncorrected and best-corrected distant visual acuity, manifest refraction, keratometric measurements, and anterior corneal higher-order aberrations (HOAs). Follow-up examinations were performed at 1 week, 1 month, and 6 months. Statistical analysis incorporated repeated-measures ANOVA and Pearson correlation coefficients. Significant improvement in UDVA was observed at all postoperative visits (*p* < 0.001). CDVA improved significantly during the early postoperative period but not at 6 months. Mean refractive astigmatism decreased markedly from − 8.19 ± 2.50 D preoperatively to − 3.14 ± 1.55 D at 6 months postoperatively, corresponding to a mean reduction of 61.3% (*p* < 0.000). Significant reductions were also observed in spherical equivalent, Keratometric indices, total HOAs and coma aberrations. Corneal thickness and spherical aberrations showed no significant changes throughout the follow-up period. No intraoperative or postoperative complications were observed. Keraring implantation may provide meaningful improvements in visual acuity, refractive outcomes, corneal regularity, and selected higher-order aberrations in eyes with high regular astigmatism and non-ectatic corneas. Larger studies with longer follow-up are warranted to confirm these preliminary findings.

**Supplementary Information:**

The online version contains supplementary material available at 10.1038/s41598-026-57128-7.

## Introduction

 Human interaction with the surrounding environment relies heavily on a well-functioning visual system that accurately focuses images onto the retina^1^.

Astigmatism is a common refractive error characterized by unequal refractive power in different meridians of the eye, resulting in blurred or distorted vision. Patients with astigmatism may experience asthenopia, visual discomfort, blurred vision, image distortion, and accommodative difficulties^2,3^.

Astigmatism is commonly classified according to its magnitude as mild (< 1.50 D), moderate (1.50–2.50 D), and high (> 2.50 D)^4^.

Increasing astigmatism has been shown to correlate with higher levels of ocular aberrations, particularly higher-order aberrations (HOAs), including coma and spherical aberrations, which may adversely affect visual quality and visual acuity. Furthermore, visual impairment resulting from uncorrected refractive errors may negatively influence quality of life and mental well-being, especially in children^5–8^.

Several treatment modalities are currently available for the correction of astigmatism, including spectacles, toric contact lenses, toric phakic intraocular lenses (IOLs), and corneal refractive surgery. Spectacles may induce image magnification and distortion, particularly in patients with high refractive errors^9^.

Although toric contact lenses minimize magnification effects, their effectiveness depends on accurate rotational alignment with the astigmatic axis^10^.

Likewise, toric phakic IOLs provide effective correction of astigmatism; however, even minor postoperative rotational misalignment may result in significant residual refractive error^11^.

Corneal refractive procedures, including photorefractive keratectomy (PRK) and laser-assisted in situ keratomileusis (LASIK), have demonstrated predictable outcomes in low-to-moderate astigmatism. However, the predictability of these procedures may decrease in eyes with high astigmatic errors, and residual refractive astigmatism may still occur following treatment^12–14^.

Intracorneal ring segments (ICRSs) have been widely used in the management of keratoconus and other corneal ectatic disorders. These implants improve visual performance by modifying corneal geometry, reducing refractive error, and improving corneal regularity. In addition, ICRS implantation has been shown to reduce corneal aberrations and improve visual acuity while maintaining the advantages of reversibility and tissue preservation^15,16^.

The potential role of ICRSs in non-ectatic corneas with high regular astigmatism has not been adequately investigated. Therefore, the present study was designed to evaluate the effectiveness of Keraring implantation for the correction of high regular astigmatism (> 3.00 D) in eyes with non-ectatic corneas that were not adequately corrected using conventional treatment modalities.

## Patients and methods

This prospective interventional case series was conducted after obtaining approval from the Institutional Review Board of the Faculty of Medicine, Sohag University, Egypt (Approval No. Soh-Med-25-4-2PD). The study was registered at ClinicalTrials.gov (Registration No. NCT06963099; registration date: May 8, 2025) and adhered to the tenets of the Declaration of Helsinki. All procedures were performed at Future Femto-Laser Center, Sohag, Egypt.

The study included 20 eyes of 20 patients with stable high astigmatism (> 3.00 D) and non-ectatic corneas. Stability of the refractive error and corneal topographic findings were confirmed for at least 12 months before surgery^17,18^.

Written informed consent was obtained from all participants before enrollment and publication of the study data.

Patients were considered eligible for Keraring implantation if they were ≥ 18 years of age, had regular corneal astigmatism ≥ 3.00 D, and demonstrated no tomographic evidence of corneal ectasia. Although the Belin–Ambrósio Enhanced Ectasia Display (BAD-D) index was not available, ectasia was excluded based on comprehensive Sirius tomographic assessment, including anterior and posterior elevation maps, pachymetric progression analysis, and the absence of focal corneal thinning or abnormal elevation patterns.

Exclusion criteria included active ocular disease (e.g., keratitis or severe dry eye disease), previous corneal surgery, pregnancy, systemic conditions affecting wound healing, and any corneal ectatic disorder, including keratoconus, pellucid marginal degeneration (PMD), or keratoglobus^19^.

All patients underwent a comprehensive ophthalmic examination, including uncorrected distance visual acuity (UDVA), corrected distance visual acuity (CDVA), manifest and cycloplegic refraction, slit-lamp biomicroscopy, and dilated fundus examination. Corneal tomography and aberrometric measurements were obtained using the Sirius anterior segment analyzer (CSO, Florence, Italy) under standardized dark-room conditions. The evaluated parameters included flat keratometry (K1), steep keratometry (K2), maximum keratometry (Kmax), mean keratometry (Kmean), and corneal thickness at the thinnest location.

Anterior corneal higher-order aberrations (HOAs) were measured over the central 4.0-mm and 6.0-mm optical zones. Based on Zernike polynomial analysis, root mean square (RMS) values were calculated for total HOAs, spherical aberration, coma, and trefoil. Only examinations with an acceptable quality status (“OK”) were included. All measurements were obtained by the same experienced technician to ensure measurement consistency and reproducibility.

All procedures were performed by the same experienced corneal surgeon (A.M.) under topical anesthesia using benoxinate hydrochloride 0.4% eye drops. Intrastromal corneal tunnels were created using a 60-kHz femtosecond laser (Abbott Medical Optics, Abbott Park, IL, USA) operating at a wavelength of 1053 nm.

The intended tunnel depth was set at 80% of the corneal thickness at the incision site. The tunnel had an inner diameter of 5.0 mm and an outer diameter of 5.9 mm. The entry incision measured 1.40 mm in length and was centered on the steepest corneal meridian. An energy setting of 1.95 J was used for both tunnel creation and entry incision formation^20^. In all cases, SI-5 model (Mediphacos Inc., Belo Horizonte, Brazil) was implanted. The segments are manufactured from polymethyl methacrylate (PMMA) and have a triangular, cross-sectional design and a 5.0- mm optical zone. Two asymmetrical segments were implanted in all eyes according to the planned surgical nomogram.

The selection of Keraring segments was based on a commercially available nomogram originally developed for keratoconus. Because no validated nomogram currently exists for the treatment of high regular astigmatism in non-ectatic corneas, this nomogram was used as the most applicable clinical reference. Segment selection was guided by the magnitude and axis of refractive astigmatism, keratometric readings, corneal thickness, and corneal topographic characteristics^21^.

All patients received antibiotic eye drops containing 0.5% moxifloxacin hydrochloride (Vigamox; Alcon Laboratories Inc., Fort Worth, Texas, USA), steroid eye drops containing 1% prednisolone acetate (Econopred Plus; Alcon Laboratories Inc.), and lubricating eye drops (Systane Ultra; Alcon Laboratories Inc.). During the first week, all topical eye drops were delivered five times daily. The antibiotic eyedrop was discontinued after 1 week, and the steroid eyedrops gradually tapered to be discontinued within 1 month in all eyes, while lubricating eye drops were applied as needed.

Patients were followed one day, one week, one month, and six months post-operative.

### Statistical analysis

Statistical analyses were performed using the Statistical Package for the Social Sciences (SPSS version 22.0; IBM Corp., Armonk, NY, USA). Quantitative variables were expressed as mean ± standard deviation (SD), whereas categorical variables were presented as frequencies and percentages.

Normality of data distribution was assessed using the Shapiro–Wilk test. Changes in visual acuity, refractive, keratometric, pachymetric, and aberrometric parameters over the follow-up period were analyzed using repeated-measures analysis of variance (ANOVA). When the overall repeated-measures ANOVA demonstrated statistical significance, Bonferroni-adjusted post hoc pairwise comparisons were performed between preoperative and postoperative visits.

Pearson correlation coefficients (r) were used to evaluate the associations between higher-order aberrations (HOAs) and clinical parameters preoperatively and at 6 months postoperatively.

Because refractive cylinder axis data was not available for all eyes, vector analysis according to the Alpins method could not be performed. Therefore, astigmatic outcomes were assessed using magnitude analysis. The absolute magnitude of manifest refractive astigmatism (cylinder power in diopters) was recorded preoperatively and at each postoperative visit.

Astigmatism reduction was calculated as follows:

Astigmatism Reduction (D) = Preoperative Cylinder − Postoperative Cylinder.

Percentage Reduction (%) = [Preoperative Cylinder − Postoperative Cylinder) / Preoperative Cylinder] × 100.

Mean astigmatism magnitude and percentage reduction were subsequently calculated for the study population.

All statistical tests were two-tailed, and a p-value < 0.05 was considered statistically significant.

## Results

The study included 20 eyes of 20 patients with a mean age of 32.11 ± 8.70 years. Twelve patients (60%) were male and eight (40%) were female. The mean preoperative spherical error was − 3.60 ± 0.94 D, the mean refractive cylinder was − 8.19 ± 2.50 D, and the mean spherical equivalent was − 7.67 ± 1.92 D^22^.

**Table 1:** Comparisons of UDVA and CDVA:

		Mean Difference	*P*-value
A) Pre and post operative UDVA			
Preoperative (1.15 ± 0.12)	1 Week (0.38 ± 0.09)	0.35	< 0.000
	1 Month (0.31 ± 0.06)	0.43	< 0.011
	6 Months (0.42 ± 0.14)	0.31	< 0.000
B) Pre and postoperative CDVA.			
Preoperative (0.38 ± 0.12)	1 Week (0.28 ± 0.06)	0.11	< 0.000
	1 Month (0.29 ± 0.06)	0.09	< 0.000
	6 Months (0.34 ± 0.09)	0.04	< 0.103

UDVA: uncorrected visual acuity (log MAR), CDVA: best corrected visual acuity (log MAR).

Table 1 shows significant improvement in uncorrected distance visual acuity (UDVA) was observed at all postoperative visits compared with preoperative values. Corrected distance visual acuity (CDVA) improved significantly at 1 week and 1 month postoperatively; however, the improvement at 6 months did not reach statistical significance.

**Table 2:** Pre-operative refractive and corneal parameters compared to post operative outcomes.

Parameter	Preoperative	1 Week	1 Month	6 Months	*P*-value
Sphere	-3.60 ± 0.94	-1.07 ± 0.31	-1.01 ± 0.97	-1.65 ± 1.10	< 0.000
Cylinder	-8.19 ± 2.50	-2.81 ± 1.09	-3.44 ± 1.34	-3.14 ± 1.55	< 0.000
SE	-7.67 ± 1.92	-1.78 ± 1.94	-2.53 ± 1.11	-3.22 ± 1.57	< 0.000
CTT	495.78 ± 28.64	493.39 ± 24.18	492.22 ± 23.33	496.28 ± 24.89	< 0.957
K1	47.27 ± 1.73	44.00 ± 1.40	44.12 ± 1.41	44.13 ± 1.54	< 0.000
K2	53.09 ± 2.69	46.41 ± 1.80	47.26 ± 2.00	47.12 ± 1.88	< 0.000
K max	57.57 ± 2.99	64.64 ± 6.34	60.09 ± 6.87	60.79 ± 5.20	< 0.003
mean K	50.17 ± 2.09	45.40 ± 1.39	45.69 ± 1.49	45.63 ± 1.53	< 0.000
RMS total	1.71 ± 0.38	1.30 ± 0.16	1.33 ± 0.16	1.33 ± 0.17	< 0.000
RMS Coma	0.96 ± 0.32	0.58 ± 0.35	0.76 ± 0.35	0.76 ± 0.31	< 0.011
RMS SA	0.53 ± 0.44	0.48 ± 0.41	0.48 ± 0.29	0.49 ± 0.29	< 0.974

Data is presented as mean ± SD, Sphere: spherical error (DS), Cylinder: cylindrical error (DC), SE: spherical equivalent, CTT: corneal thinnest location (um), K1: flat keratometry, K2: steep Keratometry, K max: maximum keratometry, mean k: mean keratometry, RMS total: total corneal high order aberrations root mean square, RMS coma: root mean square of coma high order aberrations, RMS SA: root mean square value of spherical aberration high order aberrations.

Significant reductions were observed in spherical error, refractive cylinder, spherical equivalent, K1, K2, mean keratometry, total higher-order aberrations, and coma throughout the follow-up period. Corneal thickness at the thinnest location and spherical aberration remained statistically unchanged.Table 2

Mean refractive astigmatism decreased from 8.19 ± 2.50 D preoperatively to 3.14 ± 1.55 D at 6 months postoperatively, representing a mean reduction of 5.06 D and a percentage reduction of 61.3%.

**Table 3:** Preoperative versus first Week post-operative.

Parameter	Preoperative (Mean ± SD)	1 Week (Mean ± SD)	*P*-value
Sphere	-3.60 ± 0.94	-1.07 ± 0.31	< 0.000
Cylinder	-8.19 ± 2.50	-2.81 ± 1.09	< 0.000
SE	-7.67 ± 1.92	-1.78 ± 1.94	< 0.000
CTT	495.78 ± 28.64	493.39 ± 24.18	< 0.427
K1	47.27 ± 1.73	44.00 ± 1.40	< 0.000
K2	53.09 ± 2.69	46.41 ± 1.80	< 0.000
K max	57.57 ± 2.99	64.64 ± 6.34	< 0.000
mean K	50.17 ± 2.09	45.40 ± 1.39	< 0.000
RMS total	1.71 ± 0.38	1.30 ± 0.16	< 0.000
RMS Coma	0.96 ± 0.32	0.58 ± 0.35	< 0.005
RMS SA	0.53 ± 0.44	0.48 ± 0.41	< 0.749

Data is presented as mean ± SD, Sphere: spherical error (DS), Cylinder: cylindrical error (DC), SE: spherical equivalent, CTT: corneal thinnest location (um), K1: flat keratometry, K2: steep Keratometry, K max: maximum keratometry, mean k: mean keratometry, RMS total: total corneal high order aberrations root mean square, RMS coma: root mean square of coma high order aberrations, RMS SA: root mean square value of spherical aberration high order aberrations.

**Table 4:** Preoperative versus first month post-operative.

Parameter	Preoperative (Mean ± SD)	1 Month (Mean ± SD)	*P*-value
Sphere	-3.60 ± 0.94	-1.01 ± 0.97	< 0.000
Cylinder	-8.19 ± 2.50	-3.44 ± 1.34	< 0.000
SE	-7.67 ± 1.92	-2.53 ± 1.11	< 0.000
CTT	495.78 ± 28.64	492.22 ± 23.33	< 0.111
K1	47.27 ± 1.73	44.12 ± 1.41	0.0000
K2	53.09 ± 2.69	47.26 ± 2.00	< 0.000
K max	57.57 ± 2.99	60.09 ± 6.87	< 0.120
mean K	50.17 ± 2.09	45.69 ± 1.49	< 0.000
RMS total	1.71 ± 0.38	1.33 ± 0.16	< 0.000
RMS Coma	0.96 ± 0.32	0.76 ± 0.35	< 0.046
RMS SA	0.53 ± 0.44	0.48 ± 0.29	< 0.696

Data is presented as mean ± SD, Sphere: spherical error (DS), Cylinder: cylindrical error (DC), SE: spherical equivalent, CTT: corneal thinnest location (um), K1: flat keratometry, K2: steep Keratometry, K max: maximum keratometry, mean k: mean keratometry, RMS total: total corneal high order aberrations root mean square, RMS coma: root mean square of coma high order aberrations, RMS SA: root mean square value of spherical aberration high order aberrations.

**Table 5:** Preoperative versus six-month post-operative.

Parameter	Preoperative (Mean ± SD)	6 Months (Mean ± SD)	*P*-value
Sphere	-3.60 ± 0.94	-1.65 ± 1.10	< 0.000
Cylinder	-8.19 ± 2.50	-3.14 ± 1.55	< 0.000
SE	-7.67 ± 1.92	-3.22 ± 1.57	< 0.000
CTT	495.78 ± 28.64	496.28 ± 24.89	< 0.854
K1	47.27 ± 1.73	44.13 ± 1.54	< 0.000
K2	53.09 ± 2.69	47.12 ± 1.88	< 0.000
K max	57.57 ± 2.99	60.79 ± 5.20	< 0.023
mean K	50.17 ± 2.09	45.63 ± 1.53	< 0.000
RMS total	1.71 ± 0.38	1.33 ± 0.17	< 0.000
RMS Coma	0.96 ± 0.32	0.76 ± 0.31	< 0.039
RMS SA	0.53 ± 0.44	0.49 ± 0.29	< 0.745

Data is presented as mean ± SD, Sphere: spherical error (DS), Cylinder: cylindrical error (DC), SE: spherical equivalent, CTT: corneal thinnest location (um), K1: flat keratometry, K2: steep Keratometry, K max: maximum keratometry, mean k: mean keratometry, RMS total: total corneal high order aberrations root mean square, RMS coma: root mean square of coma high order aberrations, RMS SA: root mean square value of spherical aberration high order aberrations.

Tables 3, 4 and 5 summarize the pairwise comparisons between preoperative measurements and postoperative outcomes at 1 week, 1 month, and 6 months, respectively.

**Table 6:** correlation between high order aberrations (HOA) and different refractive and corneal parameters pre-operative.

HOA Parameter	Clinical Parameter	*R*	*P*-value
RMS total	K max	0.69	< 0.001
RMS Coma	Cylinder	0.79	< 0.000
	SE	0.61	< 0.006
	K2	0.58	< 0.012
	K max	0.48	< 0.043
	BCVA	-0.54	< 0.020
RMS SA	Cylinder	0.43	< 0.074
	SE	0.48	< 0.041
	K1	0.56	< 0.015
	K2	0.60	< 0.009
	K max	0.54	< 0.020
	mean K	0.62	< 0.006
	UCVA	-0.60	< 0.008
	BCVA	-0.59	< 0.009

r : Correlation Coefficient, Sphere: spherical error (DS), Cylinder: cylindrical error (DC), SE: spherical equivalent, K1: flat keratometry, K2: steep Keratometry, K max: maximum keratometry, mean k: mean keratometry, RMS total: total corneal high order aberrations root mean square, RMS coma: root mean square of coma high order aberrations, RMS SA: root mean square value of spherical aberration high order aberrations, UCVA: uncorrected visual acuity (log MAR), BCVA: best corrected visual acuity (log MAR).

Preoperatively, total HOA demonstrated a significant positive correlation with Kmax. Coma aberration showed significant positive correlations with refractive cylinder, spherical equivalent, K2, and Kmax, whereas spherical aberration correlated significantly with spherical equivalent, K1, K2, Kmax, and mean keratometry. Significant negative correlations were also observed between visual acuity parameters and selected HOA components. Table 6

**Table 7:** Correlation between postoperative HOAs and clinical parameters at 6 Months:

`Parameter	Total HOA *r* (*p*)	Spherical aberration *r* (*p*)	Coma *r* (*p*)
CDVA	0.367 (0.134)	0.606 (0.008)	0.343 (0.163)
UDVA	0.379 (0.121)	−0.002 (0.993)	0.274 (0.270)
SE	0.246 (0.325)	−0.217 (0.387)	0.399 (0.101)
R-Cylinder	0.127 (0.616)	−0.346 (0.160)	0.006 (0.982)
K1	0.088 (0.729)	0.270 (0.279)	−0.009 (0.971)
K2	−0.065 (0.797)	0.327 (0.185)	0.032 (0.899)
Mean K	−0.066 (0.794)	0.271 (0.276)	−0.035 (0.890)
Kmax	0.316 (0.201)	−0.293 (0.237)	0.267 (0.284)
C-Cylinder	0.067 (0.791)	−0.316 (0.201)	0.109 (0.666)

Significant correlations (*p* < 0.05) are present for Astigmatism HOA and SA.

At 6 months postoperatively, total HOA did not show significant correlations with any of the evaluated clinical or corneal parameters. Astigmatism-related HOA demonstrated a significant positive correlation with CDVA (*r* = 0.606, *p* = 0.008), whereas no significant correlations were observed with UDVA, refractive, keratometric, or corneal astigmatism parameters. Similarly, spherical aberration showed no significant correlations with the studied variables.Table 7

**Table 8:** Changes in corrected distance visual acuity (CDVA):

Changes in CDVA	Number of Eyes
Gained ≥ 2 Lines	4
Gained 1 Line	7
Unchanged	6
Lost 1 Line	3
Lost ≥ 2 Lines	0

At 6 months, four eyes (20%) gained two or more lines of CDVA, seven eyes (35%) gained one line, six eyes (30%) remained unchanged, and three eyes (15%) lost one line. Importantly, no eye lost two or more lines of CDVA. Table 8

The efficacy index was 1.00 at 1 week, 1.17 at 1 month, and 0.90 at 6 months. The corresponding safety indices were 1.26, 1.21, and 1.10, respectively. No intraoperative or postoperative complications, including segment migration, extrusion, infectious keratitis, stromal haze, or the need for repositioning or explantation, were observed during the follow-up period.

**Fig. 1:**
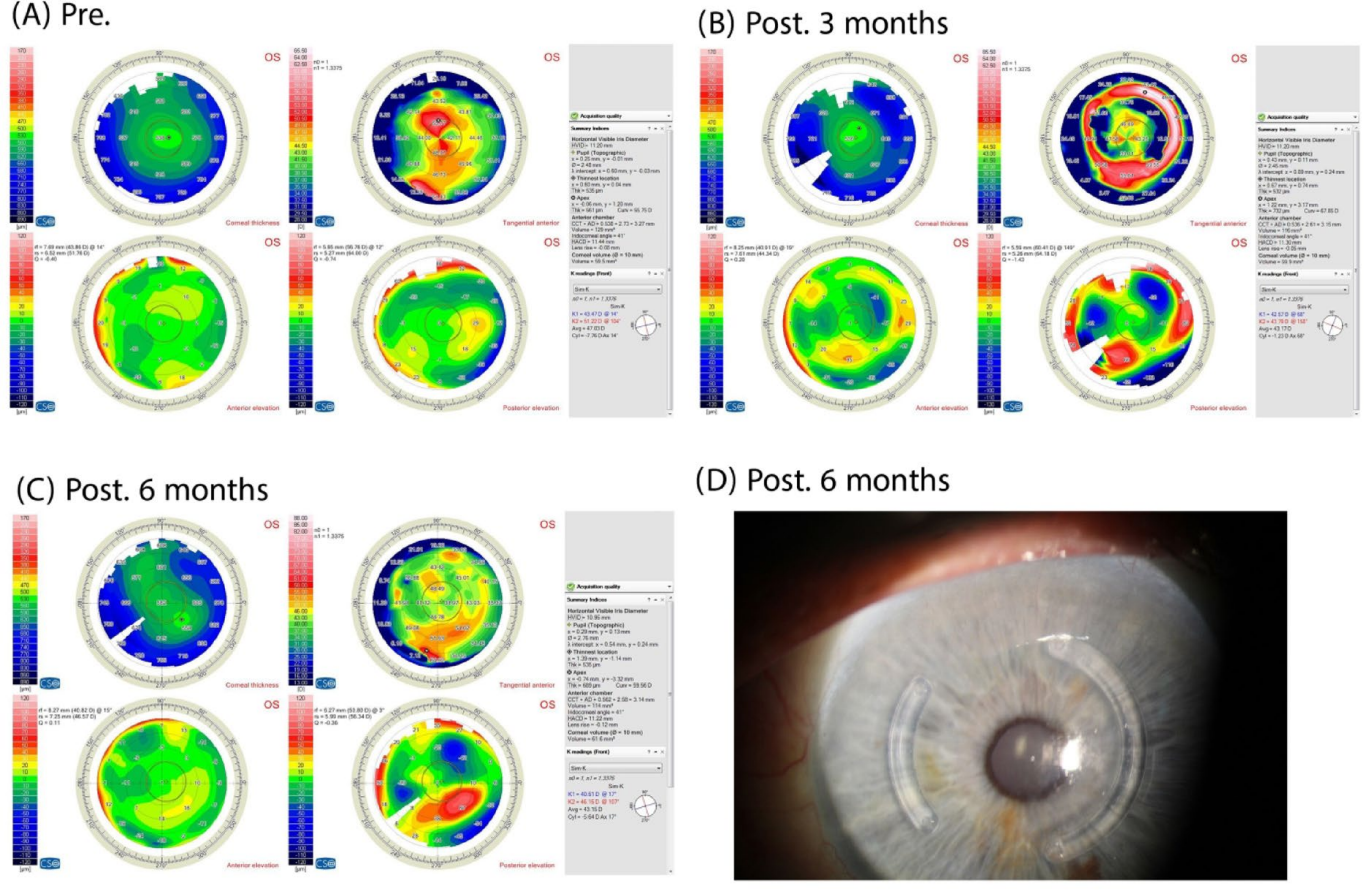
**A**. Preoperative corneal tomography (OS): Preoperative maps demonstrate high astigmatism with asymmetric corneal steepening on the tangential map, associated with mild anterior and posterior elevation variations. Pachymetry distribution appears relatively regular, with no evidence of focal thinning or tomographic features suggestive of ecstatic disease. Figure 1**B**. Postoperative 3-month tomography (OS): The maps demonstrate early corneal regularization, with reduction of the steep hemi-meridian and improved symmetry of the anterior curvature. Posterior elevation also shows decreased asymmetry, indicating early biomechanical remodeling following Keraring implantation. Figure 1**C**. Postoperative 6-month tomography (OS): Further corneal smoothing is observed, with enhanced regularity of both anterior and posterior surfaces. Astigmatism magnitude is reduced, and the tangential map shows a more uniform pattern, reflecting stable remodeling and improved optical profile. Figure 1**D**. Postoperative 6-month slit-lamp photograph (OS): Slit-lamp microscopy confirms proper centration of the Keraring segments, clear implantation channels, and absence of complications such as migration, extrusion, or stromal haze.

**Fig. 2:**
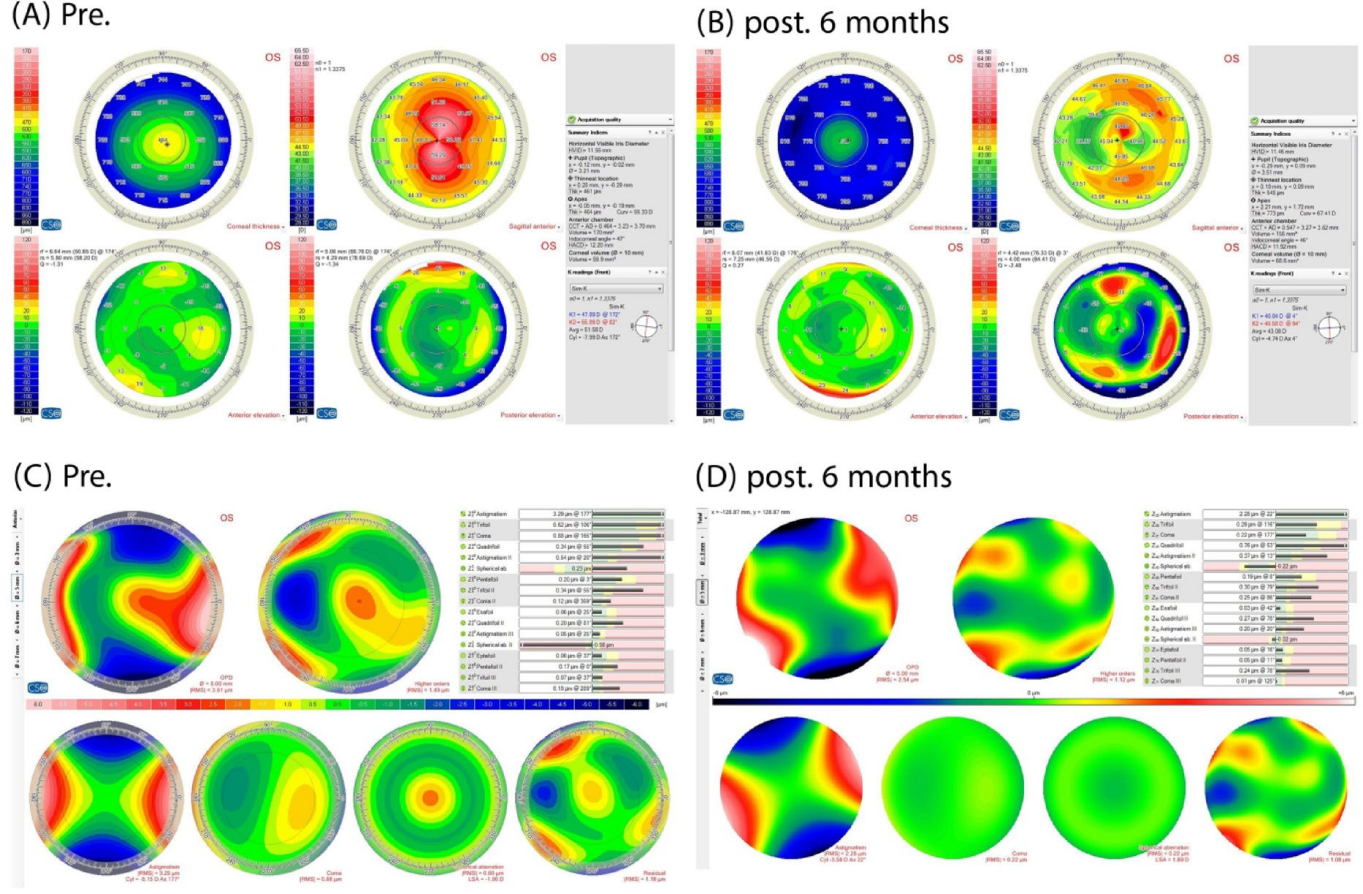
**A**. Preoperative tomography (OS): Preoperative sagittal and elevation maps demonstrate high astigmatism with asymmetric corneal curvature and mild surface elevation variations. Pachymetry shows a relatively regular thickness distribution, with no evidence of focal thinning or tomographic features suggestive of ectatic disease. 2**B**. Postoperative 6-month tomography (OS): The cornea exhibits improved curvature symmetry and reduction in astigmatic asymmetry. Elevation maps demonstrate a smoother anterior and posterior profile, consistent with corneal regularization following ring implantation.  2**C**. Preoperative aberrometry (OS): Higher-order aberration analysis shows increased coma and trefoil components, consistent with optical distortion associated with high astigmatism. 2**D**. Postoperative 6-month aberrometry (OS): Aberrometric evaluation demonstrates a reduction in total higher-order aberrations, particularly coma and trefoil components, indicating improved optical quality and corneal uniformity.

**Fig. 3:**
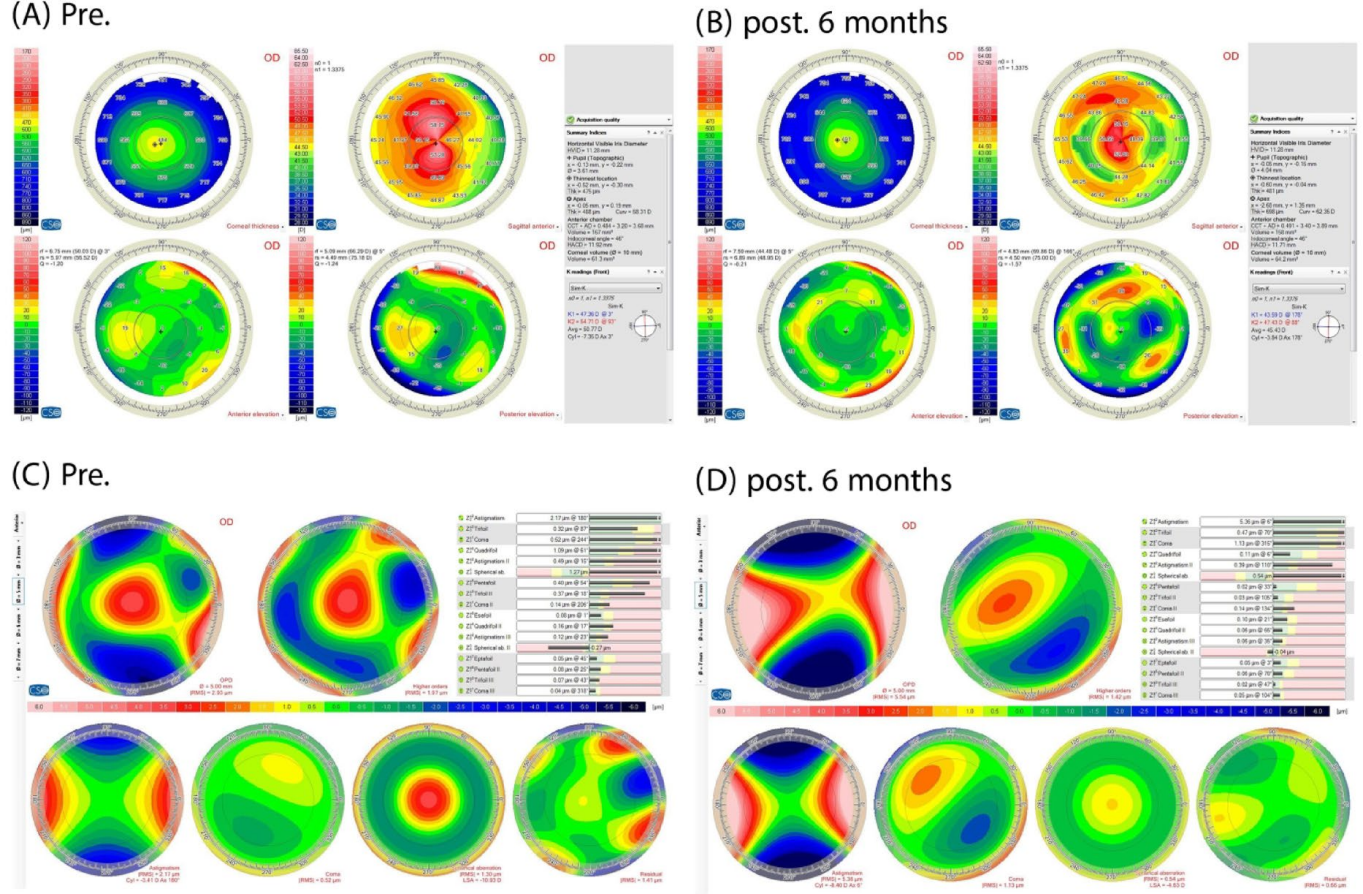
**C**. Preoperative aberrometry (OD): HOA maps reveal increased coma and trefoil aberrations, indicating optical distortion associated with high astigmatism in non-ectatic corneas. **D**. Postoperative 6-month aberrometry (OD): Postoperative aberrometric evaluation demonstrates a reduction in total higher-order aberrations, particularly coma, indicating improved visual quality and enhanced optical performance following Keraring implantation.

## Discussion

High astigmatism remains one of the most challenging refractive conditions in clinical practice, as achieving optimal visual quality and patient satisfaction after correction is often difficult. Patients with high astigmatism frequently experience reduced visual quality, image distortion, and functional visual impairment that may adversely affect daily activities. In children, uncorrected refractive errors may also negatively influence visual development and overall well-being.

Modern refractive procedures, including femtosecond laser-assisted in situ keratomileusis (FS-LASIK) and small-incision lenticule extraction (SMILE), have become established options for the correction of regular astigmatism in appropriately selected patients. However, in cases of high astigmatism or borderline corneal characteristics, laser-based procedures may be less predictable or contraindicated. In such situations, intracorneal ring segments may provide a tissue-sparing and potentially reversible alternative^23,24^.

Intracorneal ring segments (ICRSs) were initially developed for the correction of low myopia. Their indications have subsequently expanded to include a variety of corneal disorders, such as keratoconus, post-keratoplasty astigmatism, post-refractive surgery ectasia, pellucid marginal degeneration, and other forms of corneal irregularity. Several nomograms have been proposed to optimize ICRS implantation based on refractive error, corneal topography, and aberrometric characteristics. Their reversibility, long-term stability, and favorable safety profile further support their clinical application^25^.

In the present study, significant improvements were observed in both uncorrected and corrected distance visual acuity following Keraring implantation. These visual gains were accompanied by significant reductions in refractive cylinder, spherical equivalent, keratometric values, and selected higher-order aberrations. Mean refractive astigmatism decreased by 61.3% at 6 months postoperatively, suggesting that Keraring implantation may effectively reduce high regular astigmatism in selected non-ectatic corneas.

Two principal mechanisms have been proposed to explain the effect of intracorneal ring segments (ICRSs) on corneal shape. The first suggests that ICRSs act as spacer elements between corneal lamellae, producing an arc-shortening effect that leads to central corneal flattening. The second mechanism is based on Barraquer’s thickness law, which states that the addition of tissue to the corneal periphery or removal of tissue from the central cornea results in corneal flattening^26,27^.

Previous studies have demonstrated that ICRS implantation can reduce corneal astigmatism, improve corneal curvature, and enhance visual performance^28,29^.

Consistent with these findings, the present study showed significant reductions in refractive cylinder and keratometric values throughout the follow-up period. Significant flattening was observed in K1, K2, and mean keratometry. In contrast, Kmax demonstrated a postoperative increase despite the overall reduction in corneal curvature. Because Kmax represents a localized point measurement rather than a global corneal parameter, this finding may reflect localized corneal remodeling induced by the implanted segments rather than a true increase in overall corneal steepening.

Analysis of higher-order aberrations revealed significant postoperative reductions in total HOAs and coma aberrations, indicating an improvement in corneal optical quality following Keraring implantation. These findings are clinically relevant because coma aberration is one of the major contributors to visual distortion, ghosting, and reduced image quality in eyes with high astigmatism. In contrast, spherical aberration did not demonstrate a significant postoperative change. This finding is consistent with previous reports suggesting that intracorneal ring implantation may alter corneal asphericity without necessarily reducing spherical aberration^30^.

Kerarings have been shown to improve corneal symmetry, restore a more physiologic corneal contour, and redistribute corneal biomechanical forces, thereby promoting corneal regularization and reducing higher-order aberrations^31,32^.

The use of two asymmetric segments may further enhance corneal regularization and optimize the flattening effect across different meridians. This mechanism may contribute to the observed reduction in coma aberrations, which are strongly associated with symptoms such as ghosting, monocular diplopia, and visual blur^33,34^.

In the present study, preoperative HOA measurements demonstrated significant associations with both refractive and keratometric parameters. Higher levels of aberrations were associated with greater corneal irregularity and poorer visual performance. These findings are consistent with previous reports demonstrating that elevated HOAs contribute to retinal image degradation and reduced visual quality, particularly in eyes with significant refractive errors and corneal irregularities^35,36^.

Interestingly, postoperative total HOAs and coma aberrations did not demonstrate significant correlations with most postoperative clinical parameters. However, analysis of individual aberration components revealed spherical aberration demonstrated a significant association with CDVA. These findings suggest that individual aberration components may influence postoperative visual performance differently from total HOA measurements. The relationship between corneal optics and visual outcomes after Keraring implantation is likely multifactorial and may be influenced by epithelial compensation, stromal remodeling, and individual biomechanical responses^37,38^.

Recently, allogeneic intrastromal corneal implantation techniques have emerged as a potential alternative for corneal reshaping and visual rehabilitation. Although these approaches may offer advantages in terms of biological integration and tissue compatibility, their availability remains limited, and long-term clinical evidence is still evolving. In comparison, Keraring implantation remains a widely available, established, reversible, and relatively safe technique for the management of corneal refractive abnormalities^39^.

In conclusion, Keraring implantation was associated with significant improvements in visual acuity, refractive outcomes, corneal curvature, and selected higher-order aberrations in eyes with high regular astigmatism and non-ectatic corneas. The procedure resulted in substantial reduction of refractive cylinder and improvement of corneal regularity without significant safety concerns during the study period.

These findings suggest that Keraring implantation may represent a useful therapeutic option in selected patients who are not ideal candidates for conventional corneal refractive procedures. Nevertheless, larger prospective studies with longer follow-up periods are required to confirm these preliminary results and further refine patient selection criteria.

### Limitations

The present study has several limitations. First, the relatively small sample size and the absence of a control group limit the generalizability of the findings and preclude direct comparisons with other refractive treatment modalities. Second, the follow-up period was limited to 6 months and therefore may not fully reflect the long-term stability of the observed outcomes.

In addition, segment selection was based on a keratoconus-derived nomogram, as no validated nomogram is currently available for Keraring implantation in non-ectatic corneas with high regular astigmatism. Although this approach provided a practical clinical framework, it may not represent the optimal strategy for this specific patient population.

Another limitation is the absence of complete refractive axis data for all eyes, which precluded vector analysis using the Alpins method and prevented calculation of indices such as target-induced astigmatism (TIA), surgically induced astigmatism (SIA), and the difference vector. Finally, interindividual variability in corneal biomechanics and wound-healing responses may have influenced postoperative outcomes.

## Supplementary Information

Below is the link to the electronic supplementary material.

Supplementary Material 1

Supplementary Material 2

## Data Availability

All data generated or analyzed during this study are included in this published article.
