# Supplementary material for: Outcomes of keraring implantation for high regular astigmatism in non-ectatic corneas: A prospective pilot case series
Source: Sci Rep. 2026 Jun 17;16:18859. doi: 10.1038/s41598-026-57128-7 (PMC13276257; doi:10.1038/s41598-026-57128-7)
Supplement: Supplementary file 1 — Supplementary Material 1 [file 41598_2026_57128_MOESM1_ESM.docx]

# WHO-Style Clinical Trial Protocol

Study Title: Use of Kerarings in Management of Non Ectatic Corneas with High Cylinder

## 1. General Information

Short Title: Kerarings in Non-Ectatic High Cylinder

Trial Registration: ClinicalTrials.gov Identifier NCT06963099; date of first registration: 08/05/2025.

Sponsor / Coordinating Centre: Future Femto-laser Center, Sohag, Egypt, in collaboration with the Ophthalmology Department, Faculty of Medicine, Sohag University, Egypt.

Principal Investigator: Amr Mounir, MD, PhD, Assistant Professor of Ophthalmology, Sohag University, Egypt.

Co-Investigators: Elshimaa A Mateen Mossa, MD, PhD; Alaa Mahmoud, MD, PhD.

Study Design: Prospective, single-arm interventional clinical case series.

Study Location: Future Femto-laser Center, Sohag, Egypt.

## 2. Background and Rationale

High regular corneal astigmatism (>3.00 diopters) in non-ectatic eyes represents a challenging refractive problem. Conventional approaches including spectacles, toric contact lenses, phakic toric intraocular lenses, and corneal refractive surgery may fail to provide satisfactory correction in cases with very high cylindrical error. Spectacles can cause distortion and meridional magnification; toric contact lenses depend on precise rotational stability; and toric IOLs are sensitive to misalignment. Excimer laser–based procedures are most predictable in low to moderate astigmatism, leaving residual refractive error in more extreme cases.

Intrastromal corneal ring segments (ICRS), such as Kerarings, have been widely used in corneal ectatic disorders, including keratoconus, pellucid marginal degeneration, and post-refractive surgery ectasia. They act by altering corneal curvature, flattening steep areas, and reducing high and irregular astigmatism. Their reversibility, extra-ocular placement, and relative safety profile make them a promising modality.

However, the role of Kerarings in non-ectatic corneas with high regular astigmatism has been less extensively explored. This study addresses this gap by prospectively evaluating femtosecond laser–assisted implantation of SI-5 Kerarings in non-ectatic corneas with high regular cylinder, focusing on visual, refractive, keratometric, and aberrometric outcomes.

## 3. Objectives

Primary Objective:

- To evaluate the effectiveness of femtosecond laser–assisted Kerarings implantation in reducing high regular astigmatism (>3.00 D) and improving uncorrected visual acuity (UCVA) and best-corrected visual acuity (BCVA) in eyes with non-ectatic corneas.

Secondary Objectives:

- To assess changes in corneal keratometric indices (K1, K2, Kmax, mean K) after Kerarings implantation.
- To evaluate changes in anterior corneal higher-order aberrations (total RMS HOAs, coma, spherical aberration).
- To explore correlations between preoperative HOAs and refractive/keratometric parameters.
- To document any intraoperative or postoperative complications related to the procedure.

## 4. Study Design

This is a prospective, single-center, single-arm interventional case series including 20 eyes of 20 patients with high regular corneal astigmatism and non-ectatic corneas. All participants undergo standardized femtosecond-assisted Kerarings implantation with a follow-up period of 6 months. There is no control or comparison group. The study is primarily exploratory and hypothesis-generating.

## 5. Study Population

Target population: Adults with high regular corneal astigmatism and normal, non-ectatic corneas.

Sample size: 20 eyes of 20 patients.

### 5.1 Inclusion Criteria

- Age ≥ 18 years.
- Regular corneal astigmatism ≥ 3.00 diopters.
- Non-ectatic corneas confirmed by tomography: normal topographic patterns, thinnest corneal location > 450 µm, absence of clinical/tomographic signs of keratoconus, pellucid marginal degeneration, keratoglobus, or other ectatic diseases.
- Clear central cornea with no visually significant opacity.
- Ability and willingness to provide written informed consent and comply with study visits.

### 5.2 Exclusion Criteria

- Any form of corneal ectasia (keratoconus, pellucid marginal degeneration, keratoglobus, post-refractive ectasia).
- Previous corneal surgery (keratoplasty, LASIK, PRK, RK, or other corneal refractive procedures).
- Active ocular surface or intraocular inflammatory disease (e.g., keratitis, severe dry eye, uveitis).
- Significant cataract or retinal disease that may affect visual acuity.
- Pregnancy or breastfeeding.
- Systemic diseases known to impair wound healing (e.g., uncontrolled diabetes, connective tissue diseases).
- Inability to attend scheduled follow-up visits.

## 6. Study Procedures

### 6.1 Preoperative Assessment

At baseline, all participants undergo a comprehensive ophthalmic examination including:
- UCVA and BCVA (LogMAR).
- Manifest and cycloplegic refraction.
- Slit-lamp biomicroscopy of the anterior segment.
- Intraocular pressure measurement.
- Dilated fundus examination using a Volk 78D lens.
- Corneal tomography using Pentacam (OCULUS Optikgeräte GmbH, Wetzlar, Germany), including K1, K2, Kmax, mean K, and thinnest corneal location.
- Anterior corneal higher-order aberrations (HOAs) measured over 4.0- and 6.0-mm optic zones, with calculation of total RMS HOAs, coma, spherical aberration, and trefoil based on Zernike coefficients.

### 6.2 Surgical Intervention

All procedures are performed by the same experienced corneal surgeon under topical anesthesia (benoxinate hydrochloride 0.4%). A femtosecond laser (60-kHz infrared neodymium glass, 1053 nm, Abbott Laboratories Inc., USA) is used to create intrastromal corneal channels with the following parameters:
- Intended tunnel depth: 80% of total corneal thickness at the site of incision.
- Inner diameter: 5.0 mm.
- Outer diameter: 5.9 mm.
- Entry cut length: 1.40 mm; thickness: 1.0 mm.
- Incision axis: aligned with the steepest meridian according to corneal topography.
- Ring and entry cut energy: 1.95 J.

Kerarings SI-5 segments (Mediphacos Inc., Belo Horizonte, Brazil), composed of PMMA with a triangular cross-section and a 5.0 mm optical zone, are implanted through the tunnel. Two non-symmetrical segments are used in each eye, according to the degree of cylinder and topographic pattern, following the manufacturer’s nomogram. Proper centration and positioning are confirmed at the slit-lamp postoperatively.

### 6.3 Postoperative Treatment and Follow-up

Postoperative topical regimen:
- 0.5% moxifloxacin hydrochloride eye drops (Vigamox) five times daily for 1 week.
- 1% prednisolone acetate eye drops (Econopred Plus) five times daily for 1 week, then tapered and discontinued over 1 month.
- Lubricating eye drops (Systane Ultra) as needed.

Patients are instructed to avoid eye rubbing, adhere to drop regimen, and report any pain, redness, photophobia, or visual loss.

Follow-up visits are scheduled at:
- Day 1: slit-lamp examination, UCVA, assessment of ring position and early complications.
- Week 1: UCVA, BCVA, refraction, slit-lamp, intraocular pressure.
- Month 1: UCVA, BCVA, refraction, Pentacam tomography, HOAs.
- Month 6: UCVA, BCVA, refraction, Pentacam tomography, HOAs, and final outcome assessment.

## 7. Outcome Measures

Primary Outcomes:

- Change in UCVA (LogMAR) from baseline to 6 months.
- Change in BCVA (LogMAR) from baseline to 6 months.
- Change in refractive astigmatism (cylinder, DC) from baseline to 6 months.

Secondary Outcomes:

- Change in spherical equivalent (SE).
- Change in keratometric parameters (K1, K2, Kmax, mean K).
- Change in anterior corneal HOAs: total RMS, coma, spherical aberration.
- Correlation between preoperative HOAs and clinical parameters (cylinder, SE, Kmax, etc.).
- Incidence of any intraoperative or postoperative complications.

## 8. Sample Size

A total of 20 eyes of 20 patients are included. The study is exploratory and descriptive; therefore, no formal power calculation was performed. The sample size is considered adequate to demonstrate trends in visual, refractive, keratometric, and aberrometric changes and to assess the safety profile in this specific population.

## 9. Randomization and Blinding

This is a single-arm interventional case series without randomization or a control group. Therefore, random sequence generation, allocation concealment, and masking/blinding procedures are not applicable. All participants receive the same intervention according to a standardized protocol.

## 10. Data Collection and Management

Data collection is performed using standardized case report forms (CRFs). Variables collected include demographic data, UCVA, BCVA, manifest and cycloplegic refraction, keratometric parameters, HOAs, and any adverse events.

Each participant is assigned a unique study ID code. Identifiable personal data are stored separately from clinical data. Electronic databases are password-protected and accessible only to the principal investigator and authorized study staff. Data will be stored for at least five years after study completion, in accordance with institutional and regulatory requirements.

## 11. Statistical Analysis Plan

Data will be analyzed using SPSS (version 22.0 or later). Continuous variables will be summarized as mean ± standard deviation. The following analyses are planned:
- Repeated-measures ANOVA to compare preoperative and postoperative values of UCVA, BCVA, refractive error, keratometry, and HOAs at 1 week, 1 month, and 6 months.
- Where relevant, paired t-tests may be used to compare baseline values with specific postoperative time points.
- Pearson correlation coefficients (r) will be calculated to evaluate relationships between HOAs and clinical parameters (cylinder, SE, K1, K2, Kmax, mean K, UCVA, BCVA).
- A p-value < 0.05 will be considered statistically significant.

## 12. Quality Assurance and Monitoring

All examinations are performed by the same experienced technician using the same calibrated equipment (Pentacam). Only measurements with “OK” quality indices are accepted. All surgeries are performed by a single experienced corneal surgeon to reduce inter-surgeon variability. The principal investigator periodically reviews CRFs and source data to ensure accuracy and completeness.

## 13. Ethical Considerations

The study received ethical approval from the Institutional Review Board (IRB) of Sohag Faculty of Medicine, Sohag University, Egypt (approval number as stated in the manuscript). The trial adheres to the principles of the Declaration of Helsinki. All participants receive detailed verbal and written information about the nature of the study, potential benefits, and possible risks. Written informed consent is obtained before any study procedures are performed.

Participants retain the right to withdraw from the study at any time without prejudice to their standard clinical care.

## 14. Potential Risks and Benefits

Potential risks include typical risks associated with intrastromal corneal ring segment implantation: infection, inflammation, ring migration or extrusion, stromal haze, over- or under-correction, glare, halos, and reduced visual quality. These risks are minimized through adherence to aseptic technique, careful surgical planning, and close postoperative monitoring.

Potential benefits for participants include reduction in high astigmatism, improvement in visual acuity, and improved quality of life. The study may also contribute valuable knowledge regarding the use of Kerarings in non-ectatic high-cylinder eyes.

## 15. Dissemination of Results

The results of this study will be submitted for publication in peer-reviewed journals (including Scientific Reports) and may be presented at national and international ophthalmology conferences. No identifiable patient information will be disclosed in any dissemination. Participants may request a summary of the study results once the study is completed and published.

## 16. Protocol Version and Timeline

Protocol version: 1.0

Estimated timeline:
- Study initiation and patient recruitment: as per IRB approval and trial registration.
- Recruitment period: until 20 eligible patients are enrolled.
- Follow-up duration: 6 months after surgery for each participant.
- Data analysis and manuscript preparation: after completion of follow-up for all participants.
